# Associations of Demographic, Lifestyle, and Clinical Factors With the Presence of Dupuytren Disease: Results from the Lifelines Cohort Study

**DOI:** 10.1016/j.jhsg.2025.100786

**Published:** 2025-07-24

**Authors:** Michel F.N. Noordman, Sophie A. Riesmeijer, Paul M.N. Werker, Ilja M. Nolte

**Affiliations:** ∗ University of Groningen, University Medical Center Groningen, Department of Plastic Surgery, Groningen, The Netherlands; † University of Groningen, University Medical Center Groningen, Department of Epidemiology, Groningen, The Netherlands

**Keywords:** Association, Dupuytren disease, Hierarchical modeling, Lifelines, Risk factors

## Abstract

**Purpose:**

Many risk factors have been associated with Dupuytren disease (DD), but their contribution is still unclear. Therefore, we aimed to investigate the associations of a wide range of risk factors with the presence of DD in Lifelines, an ongoing prospective population-based cohort study with >165,000 participants initiated in 2006.

**Methods:**

The presence of DD was determined through questionnaires by self-reported doctor’s diagnosis. The association between demographic, lifestyle, and clinical factors and DD was analyzed using logistic regression adjusted for age, age^2^, and sex. If *P* < .25, the variable was selected for inclusion in multivariable logistic regression models. Related risk factors were grouped into blocks to overcome multicollinearity. Stepwise hierarchical modeling was applied. Nested models were compared using log-likelihood ratio tests. Sensitivity analysis using controls >55 years was performed to assess the robustness of the findings.

**Results:**

Overall, 1,320 (2.1%) Lifelines participants reported to have DD. Age, age^2^, and sex accounted for 7.8% of the variability observed in DD risk. Other risk factors for DD were (osteo)arthritis, anti-inflammatory or antirheumatic products, high-density lipoprotein levels, triglyceride levels, alcohol use, and diabetes and diabetes medication, while anthropometric measures of adiposity were negatively associated with DD. Their contribution was relatively small, with the explained variance increasing only to 8.76%.

**Conclusions:**

Older age and male sex were the predominant factors increasing DD risk, but anthropometric measures of adiposity, (osteo)arthritis, anti-inflammatory and antirheumatic drugs, high-density lipoprotein levels, triglyceride levels, alcohol use, diabetes and diabetes medication also contributed significantly to the final risk model for DD. In particular, the joint related factors are of interest because previous evidence for these risk factors was inconclusive.

**Clinical relevance:**

Our risk model presents an opportunity for prevention of DD. Future studies should elucidate the role of rheumatoid arthritis in DD. Risk models may possibly enable the creation of accurate individual risk profiles of DD leading to optimization of care.

Dupuytren disease (DD) is a fibrotic disorder of the hands in which the palmar fascia thickens and contracts, causing permanent flexion contractures of mostly the little and ring fingers.^1^ Depending on the region and the studied population, DD prevalence can range from 0.6% to 31.6% with differences in DD prevalence between ethnicities being partly explained by population differentiation, with highest prevalences in northwestern Europe.^2^, ^3^, ^4^, ^5^, ^6^ Moreover, the prevalence of DD increases with age.^3^ Although the flexion contractures can be reduced with treatments such as percutaneous needle fasciotomy, *Clostridium histolyticum*-derived collagenase, or limited fasciectomy, this does not cure the disease. Recurrence rates are high varying from 21% to 85% 5 years after surgery, depending on the treatment type and the definition of recurrence used.^7^ The right timing and type of treatment is integral to minimize the risk and impact of recurrence. Currently, however, this choice is based on DD diathesis at best, often before knowing about the full extent or development of a patient’s DD phenotype.^8^^,^^9^ Therefore, it is essential to uncover the factors driving DD risk and eventually DD recurrence to optimize and personalize clinical decision making.

Previous research has shown that DD is a complex multifactorial disorder that is caused by both genetic and nongenetic factors.^10^ Suggested predisposing factors include advanced age, male sex, and family history of DD.^11^ Additionally, medical conditions like hyperlipidemia, frozen shoulder, liver disease, epilepsy, and diabetes mellitus have been suggested to positively associate with DD, as well as lifestyle factors such as heavy alcohol use, smoking, and increased manual work in profession or leisure.^9^^,^^12^, ^13^, ^14^, ^15^ Contrastingly, higher body mass index (BMI) was shown to be negatively correlated with and causally associated with a lower DD.^16^^,^^17^ However most of the studies that investigated the aforementioned associations, have important limitations.^11^ First, there was large heterogeneity in terms of study design, effect size measurements, and the use of definitions of possible risk factors. Second, some studies had small sample sizes that made it difficult to draw definite conclusions, or did not adjust for important confounders. Other studies were confined to the inclusion of specific age groups only. To our knowledge, only one study using UK Biobank data has managed to overcome these methodological challenges to date.^18^ The study found strong associations of diabetes and diabetes related microvascular complications, BMI, lipid levels, alcohol, smoking, hypertension, and respiratory disease with DD.^18^ However, some possible risk factors were left unexplored, especially certain medication types. Therefore, in this study, we investigated the associations of demographic, lifestyle, and clinical factors with the presence of DD within the large multigenerational Lifelines Cohort Study.^19^, ^20^, ^21^

## Materials and Methods

### Study design and patient population

In this study, we used data from the Lifelines Cohort Study, which has been extensively described before.^19^, ^20^, ^21^ Lifelines is a multi-disciplinary prospective population-based cohort study examining in a unique three-generation design the health and health-related behaviours of 167,729 persons living in the North of the Netherlands. It employs a broad range of investigative procedures in assessing the biomedical, socio-demographic, behavioural, physical and psychological factors which contribute to the health and disease of the general population, with a special focus on multi-morbidity and complex genetics.^21^ Baseline data of participants were collected between 2006 and 2013 with ages ranging from 6 months to 93 years. All participants visited a Lifelines research site for a baseline physical examination by trained medical personnel using standardized protocols. Fasting blood samples and 24-hour urine were collected as well as information regarding demographic characteristics, lifestyle, psychosocial factors, health status, and medication use by extensive questionnaires. Participants received follow-up questionnaires every 1.5 years and invitations for follow-up physical examinations every 5 years. Signed informed consent forms were acquired from all participants. The Lifelines Cohort Study is conducted according to the principles of the Declaration of Helsinki, in accordance with the research code of University Medical Center Groningen. The Lifelines protocol was approved by the UMCG medical ethical committee under number 2007/152. For the present study, we adhered to the Strengthening the Reporting of Observational Studies in Epidemiology guidelines.

### Measurements

The presence of DD was determined using two questionnaires distributed approximately 10 years after baseline (visit 3a) and approximately 12 years after baseline (3b). Data regarding DD were not available before these timepoints. Participants who answered “yes” to the question “Were you ever diagnosed with Dupuytren disease by a doctor?” were regarded as cases. In cases where DD was self-reported at visit 3b but not at visit 3a, the participant was classified as having DD (incident case; n = 141). Participants who reported DD at visit 3a but not at visit 3b were excluded (inconsistent reporting; n = 69). Participants who answered “no” in both questionnaires were selected as controls. Other DD data such as family history were not available.

Regarding risk factors, we included demographic, lifestyle, and clinical factors (Table S1, available online on the Journal’s website at https://www.jhsgo.org). Demographic factors were age and biological sex. Lifestyle factors included body mass index (BMI), waist and hip circumference, waist-to-hip ratio, alcohol use (not/moderate/heavy), and smoking (packyears and smoking categories never/ex-smoker/current-smoker). Alcohol use was defined according to the National Health Service criteria.^22^ Therefore, heavy drinking was defined as drinking >14 units of alcohol/week. Drinking <14 units/week but >0 was classified as moderate drinking. Packyears was defined as the equivalent of smoking one pack of 20 cigarettes a day for 1 year.

As increased prevalence of DD has been observed in patients with diabetes mellitus, we also included diabetes as a risk factor.^13^^,^^23^ Participants were classified as having diabetes mellitus in case of self-reported diabetes (type 1 or 2), use of diabetes medication, hemoglobin A1c level ≥ 6.5%, or fasting plasma glucose level ≥ 7.0 mmol/L. Participants reporting diabetes gravidarum were excluded.

Additionally, we included serum low-density lipoprotein (LDL) level, serum high-density lipoprotein (HDL) level, total cholesterol (TC) level, triglyceride level, lipemia index, apolipoprotein A1 level, apolipoprotein B100 level, systolic blood pressure (SBP), diastolic blood pressure (DBP), alanine aminotransferase level, and aspartate aminotransferase (ASAT) levels, for which reported associations with DD were still inconclusive.^11^ Serum HDL, LDL and TC levels were corrected for cholesterol medication use at baseline in order to estimate the true value without medication use: serum HDL was multiplied by 0.949, LDL was multiplied by 1.352, and TC was multiplied by 1.271.^24^ SBP and DBP were corrected for antihypertensives use by adding 15 and 10 mm Hg, respectively. Based on the adjusted values, mean arterial pressure (MAP) was calculated as follows: (2 × DBP + SBP)/3. Other health problems included in this study as risk factors for DD were rheumatoid arthritis, osteoarthritis, gallstones, hepatitis, lung fibrosis, and liver disorder (Table S1).

Since little is known about the association between medication and DD, we included several medication groups as potential risk factors for DD: diuretics, lipid modifying agents, calcium channel blockers, agents acting on the renin-angiotensin system, other antihypertensives, antithrombotic agents, diabetes drugs, immunostimulants, immunosuppressants, antigout preparations, anti-inflammatory drugs, and antirheumatic drugs. Medication types were classified according to the Anatomical Therapeutic Chemical classification system.^25^

Risk factors were grouped into blocks of related variables after careful judgment in our research group (Table S1). If possible, we obtained the data of risk factors from visit 3a or 3b. In cases where data were not available at either of these visits, data were obtained from the most recent visit before that (Table S1).

### Statistical analysis

All analyses were performed using R (version 4.3.0). Data are presented as mean ± standard deviation (SD) when normally distributed and as median and interquartile range (IQR) when skewed as assessed using QQ-plots. Counts and proportions are used for describing nominal data. Continuous variables were checked for outliers and values beyond six standard deviations from the mean were set to missing.

Each variable was tested individually for its impact on the presence of DD by univariable logistic regression models with DD as response. Because DD occurs most commonly at older age and in males, we repeated all univariable regression analyses with age, age^2^, and sex as possible confounders, with age^2^ accounting for the nonlinear relationship between age and DD presence.^3^ The interaction terms of sex with age and anthropometric traits were tested to investigate whether age and the anthropometric indices have a different effect on DD in males and females. When *P* < .25, the variable was selected for inclusion in multivariable logistic regression models, for which stepwise hierarchical modeling was applied meaning that blocks of related variables were sorted based on increasing significance of the most significant variable within the block and added in that order to the multivariable model. The log-likelihood ratio test was applied to test for significance of each added block to find the most optimal model for DD presence.

The explained variance of the models was calculated using Nagelkerke’s pseudo-R^2^ after establishing the final model and using only individuals with complete data for the variables included in this final model. The difference in R^2^ (ΔR^2^) between nested models was determined to estimate the contribution of the added block of variables.

A sensitivity analysis was performed only including controls aged over 55 years for which all of the analyses above were repeated, in order to rule out confounding by age and assess the robustness of the associations.

## Results

A total of 62,941 Lifelines participants answered the question about DD diagnosis. Of these, 1,320 (2.1%) reported a positive DD diagnosis. Fifty-seven percent of the participants with DD were men versus 41% of the controls. Participants with DD had a mean (± standard deviation [SD]) age of 64.3 ± 9.2 years. This was 55.1 ± 12.6 years for participants without DD. Further characteristics are shown in Table 1. Using only controls aged >55 years, the mean ages of participants with and without DD were 64.3 ± 9.2 and 64.5 ± 6.9 years, respectively (Table S2, available online on the Journal’s website at https://www.jhsgo.org).

**Table 1:** Characteristics Stratified for DD Patients and Controls∗

Characteristic	DD (N = 1,320)†	No DD (N = 61,621)†
***Demographic***		
Age, years	64.3 (9.2)	55.1 (12.6)
Sex (male)	746 (57%)	25,237 (41%)
***Anthropometric***		
BMI	25.9 (23.7–28.6)	26.1 (23.7–29.1)
Waist circumference	93.0 (86.0–101.0)	91.0 (82.5–100.0)
Hip circumference	100.0 (95.5–105.0)	101.0 (96.0–107.0)
Waist-to-hip ratio	0.93 (0.86–0.99)	0.89 (0.83–0.96)
***Diabetes related***		
Diabetes		
No	1228 (93.2%)	59,435 (96.5%)
Yes	90 (6.8%)	2129 (3.5%)
Drugs used in diabetes		
No	1274 (96.5%)	60,855 (98.8%)
Yes	46 (3.5%)	766 (1.2%)
HbA1c	38.0 (36.0–40.0)	37.0 (35.0–40.0)
Glucose	5.3 (5.0–5.8)	5.2 (4.9–5.6)
***Lipid related***		
LDL cholesterol‡	3.4 (0.9)	3.4 (0.9)
HDL cholesterol‡	1.5 (1.2–1.8)	1.5 (1.2–1.8)
TC‡	5.3 (1.0)	5.3 (1.0)
Triglycerides	1.1 (0.84–1.50)	1.1 (0.81–1.52)
Lipemia index	10.0 (8.0–13.0)	11.0 (9.0–13.0)
Apolipoprotein A1	1.5 (0.3)	1.5 (0.3)
Apolipoprotein B100	1.0 (0.2)	0.9 (0.2)
Lipid modifying agents		
No	1193 (90.4%)	58,437 (94.8%)
Yes	127 (9.6%)	3184 (5.2%)
Antithrombotic agents		
No	1240 (93.9%)	59,808 (97.1%)
Yes	80 (6.1%)	1813 (2.9%)
***Blood pressure related***		
SBP‡	128.0 (118.0–141.0)	124.0 (114.0–135.0)
DBP‡	77.0 (69.0–84.0)	73.0 (67.0–81.0)
MAP‡	94.0 (86.3–102.0)	90.3 (83.3–98.3)
RAAS-acting agents		
No	1193 (90.4%)	58,104 (94.3%)
Yes	127 (9.6%)	3517 (5.7%)
Calcium channel blockers		
No	1277 (96.7%)	60,655 (98.4%)
Yes	43 (3.3%)	966 (1.6%)
Diuretics		
No	1255 (95.1%)	59,793 (97.0%)
Yes	65 (4.9%)	1828 (3.0%)
Other antihypertensives		
No	1318 (>99.3%)	61,556 (99.9%)
Yes	<10 (<0.7%)	65 (0.1%)
***Smoking related***		
Packyears	3.0 (0.0–12.2)	0.3 (0.0–8.4)
Smoking		
Never	496 (37.7%)	28,194 (46.8%)
Ex	691 (52.5%)	24,147 (40.1%)
Current	129 (9.8%)	7,928 (13.2%)
***Alcohol intake***		
Alcohol		
Not	249 (18.9%)	13,773 (22.6%)
Moderate	972 (73.7%)	44,148 (72.4%)
Heavy	97 (7.4%)	3062 (5.0%)
***Joint related***		
Arthritis		
No	1229 (93.1%)	59,176 (96.0%)
Yes	91 (6.9%)	2445 (4.0%)
Osteoarthritis		
No	860 (65.2%)	48,842 (79.3%)
Yes	460 (34.8%)	12,779 (20.7%)
Anti-inflammatory and antirheumatic products		
No	1237 (93.7%)	59,290 (96.2%)
Yes	83 (6.3%)	2331 (3.8%)
Antigout preparations		
No	1314 (>99.3%)	61,459 (99.7%)
Yes	<10 (<0.7%)	162 (0.3%)
Immunostimulants		
No	1320 (>99.3%)	61,585 (99.9%)
Yes	<10 (<0.7%)	36 (0.1%)
Immunosuppressants		
No	1313 (>99.3%)	61,299 (99.5%)
Yes	<10 (<0.7%)	322 (0.5%)
***Gastrointestinal and liver related***		
Gallstones		
No	1259 (95.4%)	58,797 (95.4%)
Yes	61 (4.6%)	2824 (4.6%)
Hepatitis		
No	1294 (98.0%)	60,920 (98.9%)
Yes	26 (2.0%)	701 (1.1%)
Liver disorder		
No	1310 (>99.3%)	61,240 (99.4%)
Yes	<10 (<0.7%)	381 (0.6%)
ALAT	21.0 (16.0–28.0)	19.0 (14.0–27.0)
ASAT	24.0 (20.0–27.0)	23.0 (20.0–27.0)
***Other variables***§		
Lung fibrosis		
No	1060 (>99.2%)	46,794 (99.3%)
Yes	<10 (<0.8%)	338 (0.7%)

HbA1c, hemoglobin A1c; RAAS, renin-angiotensin-aldosterone system.

∗ Presence of DD was determined by a positive answer to the question whether he/she was diagnosed by a doctor. Controls are the participants who reported not to have a positive doctor’s diagnosis of DD.

† Continuous variables are described using mean (SD) if normally distributed or median [interquartile range] otherwise; categorical data are described as count (percentage).

‡ Adjusted for medication use (see methods).

§ The term “Other variables” refers to variables that did not fit in one of the other groups of variables.

Increasing age (odds ratio (OR) = 1.07 [95% confidence interval (CI), 1.06–1.08]; *P* < 2 × 10^–16^) and male sex (OR = 1.87 [1.68–2.09]; *P* < 2 × 10^–16^) were significantly associated with DD (Table 2). A negative association was found between age^2^ and DD (OR = 0.998 [0.998–0.999]; *P* = 1.2 × 10^–13^). Many other included factors were significantly associated with DD presence. However, after adjusting for age, age^2^, and sex, most associations disappeared. Having a higher serum HDL level (OR = 1.34 [1.16–1.55]; *P* = 5.6 × 10^–5^) was still significantly associated with DD presence. Participants who were moderate or heavy alcohol drinkers (OR=1.18 [1.02–1.36]; *P* = .024 and OR = 1.59 [1.24–2.02]; *P* = 2×10^-4^, respectively) had a higher risk of DD compared to nondrinkers. Additionally, having diabetes (OR = 1.19 [0.95–1.48]; *P* = 0.13), rheumatoid arthritis (OR = 1.34 [1.07–1.66]; *P* = .009), or osteoarthritis (OR = 1.34 [1.18–1.51]; *P* = 2.3 × 10^-6^) was positively associated with DD presence. Furthermore, we found a positive association of diabetes medication (OR = 1.61 [1.17–2.17]; *P* = .002) and anti-inflammatory and antirheumatic agents (OR = 1.46 [1.15–1.82]; *P* = .001) with DD. Conversely, a higher BMI (OR = 0.97 [0.95–0.98]; *P* = 1.9 × 10^–6^), waist circumference (OR = 0.99 [0.99–1.00]; *P* = .001), hip circumference (OR = 0.99 [0.98–1.00]; *P* = .005), waist-to-hip ratio (OR = 0.40 [0.18–0.89]; *P* = .03), SBP (OR = 0.995 [0.992–0.999]; *P* = .01), and MAP (OR=0.995 [0.990–1.000]; *P* = .045) were found to significantly decrease DD risk. DBP (OR = 0.996 [0.991–1.002]; *P* = .20), ASAT level (OR = 0.99 [0.98–1.00]; *P* = .18), triglyceride level (OR=0.93 [0.86–1.01]; *P* = .08), and (ex-)smoking (OR = 1.09 [0.97–1.23]; *P* = .16) were also included in subsequent multivariable analyses since *P* < .25. Lastly, the interaction terms waist circumference × sex (OR = 0.99 [0.98–1.00]; *P* = .02), hip circumference × sex (OR = 0.99 [0.97–1.00]; *P* = .08), waist-to-hip ratio × sex (OR = 0.15 [0.03–0.73]; *P* = .02), and BMI × sex (OR = 0.96 [0.93–0.99]; *P* = .004) showed negative effects on DD.

**Table 2:** Univariable Logistic Regression Analyses Results Without (Unadjusted OR and *P* Value) and With Correction (Adjusted OR and *P* Value) for Age, Age^2^, and Sex∗

Variable	Unadjusted		Adjusted	
	OR (95% CI)	*P* Value	OR (95% CI)	*P* Value
***Demographic - 1***				
Age	1.069 (1.0–641.075)	**<2 × 10^–16^**	N.A.	N.A.
Age	1.100 (1.089–1.111)	**<2 × 10^–16^**	N.A.	N.A.
Age^2^	0.998 (0.998–0.999)	**1.2 × 10^–13^**	N.A.	N.A.
Sex (male)	1.87 (1.68–2.09)	**<2 × 10^–16^**	N.A.	N.A.
Age × sex	N.A.	N.A.	0.998 (0.986–1.011)	0.80
***Anthropometric - 2***				
BMI	0.98 (0.97–0.99)	**.003**	0.97 (0.95–0.98)	**1.9 × 10^–6^**
BMI × sex	N.A.	N.A.	0.96 (0.93–0.99)	**.004**
Waist circumference	1.012 (1.008–1.016)	**2.5 × 10^–8^**	0.992 (0.987–0.997)	**.001**
Waist circumference × sex	N.A.	N.A.	0.99 (0.98–1.00)	**.02**
Hip circumference	0.985 (0.978–0.991)	**1.6 × 10^–6^**	0.990 (0.983–0.997)	**.005**
Hip circumference × sex	N.A.	N.A.	0.99 (0.97–1.00)	*.08*
Waist-to-hip ratio	30.8 (17.6–54.1)	**<2 × 10^–16^**	0.40 (0.18–0.89)	**.03**
Waist-to-hip ratio × sex	N.A.	N.A.	0.15 (0.03–0.73)	**.02**
***Diabetes related - 6***				
Diabetes	2.05 (1.63–2.53)	**1.3 × 10^–10^**	1.19 (0.95–1.48)	*.13*
Diabetes drugs	2.87 (2.09–3.84)	**8.9 × 10^–12^**	1.61 (1.17–2.17)	**.002**
HbA1c	1.04 (1.03–1.05)	**<2 × 10^-16^**	1.00 (0.99–1.01)	.78
Glucose	1.22 (1.15–1.28)	**2.8 × 10^–14^**	1.01 (0.95–1.07)	.67
***Lipid related - 4***				
LDL cholesterol†	1.06 (1.00–1.13)	**.04**	0.99 (0.93–1.05)	.80
HDL cholesterol†	1.09 (0.96–1.25)	*.18*	1.34 (1.16–1.55)	**5.6** × **10^–5^**
TC†	1.07 (1.02–1.13)	**.009**	1.01 (0.96–1.07)	.71
Triglycerides	1.03 (0.96–1.10)	.43	0.93 (0.86–1.01)	*.08*
Lipemia index	0.99 (0.98–1.003)	.24	1.00 (0.99–1.00)	.34
Apolipoprotein A1	1.33 (0.89–1.99)	*.16*	1.29 (0.82–2.02)	.26
Apolipoprotein B100	2.44 (1.57–3.80)	**7.6 × 10^–5^**	0.99 (0.61–1.60)	.97
Lipid modifying agents	1.95 (1.61–2.34)	**1.9 × 10^–12^**	0.94 (0.77–1.14)	.54
Antithrombotic agents	2.13 (1.68–2.66)	**1.4 × 10^–10^**	0.95 (0.74–1.20)	.68
***Blood pressure related-7***				
SBP†	1.015 (1.012–1.018)	**<2 × 10^–16^**	0.995 (0.992–0.999)	**.01**
DBP†	1.025 (1.020–1.031)	**<2 × 10^–16^**	0.996 (0.991–1.002)	*.20*
MAP†	1.023 (1.019–1.028)	**<2 × 10^–16^**	0.995 (0.990–1.000)	**.045**
RAAS-acting agents	1.76 (1.45–2.11)	**2.7 × 10^–9^**	0.92 (0.76–1.11)	.39
Calcium channel blockers	2.11 (1.53–2.85)	**2.3 × 10^–6^**	1.09 (0.78–1.48)	.59
Diuretics	1.69 (1.30–2.16)	**4.6 × 10^–5^**	0.96 (0.74–1.24)	0.78
Antihypertensives	1.44 (0.24–4.59)	.61	0.93 (0.15–2.99)	0.92
***Smoking related - 8***				
Packyears	1.019 (1.014–1.024)	**1.7 × 10^–14^**	1.000 (0.994–1.005)	.89
Smoking				
Never	Ref.	Ref.	Ref.	Ref.
Ex	1.63 (1.45–1.83)	**2.9** × **10^–16^**	1.09 (0.97–1.23)	*.16*
Current	0.92 (0.76–1.12)	.43	0.97 (0.79–1.17)	.73
***Alcohol intake - 5***				
Alcohol				
Not	Ref.	Ref.	Ref.	Ref.
Moderate	1.22 (1.06–1.40)	**.006**	1.18 (1.02–1.36)	**.024**
Heavy	1.75 (1.38–2.21)	**3.8 × 10^–6^**	1.59 (1.24–2.02)	**.0002**
***Joint related - 3***				
Arthritis	1.79 (1.43–2.21)	**1.3 × 10^–7^**	1.34 (1.07–1.66)	**.009**
Osteoarthritis	2.04 (1.82–2.29)	**<2 × 10^–16^**	1.34 (1.18–1.51)	**2.3 × 10^–6^**
Anti-inflammatory and antirheumatic drugs	1.71 (1.35–2.13)	**3.6 × 10^–6^**	1.46 (1.15–1.82)	**.001**
Antigout preparations	1.73 (0.68–3.59)	*.19*	0.86 (0.34–1.79)	.72
Immunostimulants	2.2 **×** 10^–5^ (4.2 **×** 10^–25^–0.021)	.94	3.4 **×** 10^–5^ (2.6 **×** 10^–24^ 0.026)	.94
Immunosuppressants	1.01 (0.43–1.99)	.97	0.84 (0.36–1.65)	.64
***Gastrointestinal and liver related - 9***				
Gallstones	1.00 (0.77–1.30)	.95	0.92 (0.70–1.19)	.54
Hepatitis	1.75 (1.15–2.54)	**.006**	1.13 (0.74–1.65)	.54
Liver disorder	1.23 (0.61–2.18)	.53	1.19 (0.59–2.13)	.59
ALAT	1.004 (1.000–1.008)	**.02**	0.999 (0.992–1.004)	.75
ASAT	1.01 (1.00–1.02)	*.06*	0.991 (0.98–1.00)	*.18*
***Other variables***‡				
Lung fibrosis	1.31 (0.65–2.32)	.41	0.96 (0.48–1.72)	.91

N.A., not applicable; Ref., reference.

∗ Interaction terms were only tested in models corrected for age, age^2^, and sex. Significant *P* values (*P* < .05) are indicated in bold, *P* value < .25 in italic. The number after the name of the block of variables indicates the order of significance as used for the multivariable, hierarchical modeling, with no number meaning that none of the individual variables was significant.

† Adjusted for cholesterol or blood pressure lowering medication use.

‡ The term “Other variables” refers to variables that did not fit in one of the other groups of variables.

Based on the univariable results adjusted for age, age^2^, and sex, blocks of related variables with *P* < .25 were tested hierarchically (Table S1). The base model with age, age^2^, and sex explained 7.80% of the individual differences in DD presence (Table 3). By including the four anthropometric measures and interaction terms with sex (model 2), the explained variance increased by 0.35%. Next, adding arthritis, osteoarthritis, and anti-inflammatory and antirheumatic agents explained 0.32% more of the variance. After adding HDL and triglyceride levels to model 3, the explained variance increased with 0.07%. An increase of 0.11% explained variance was achieved by adding alcohol use to model 4. Finally, diabetes and diabetes drugs caused another 0.06% increase in explained variance. Blocks 7, 8, or 9 did not improve the model. Model 6 was the final model, explaining 8.76% of the individual differences in DD presence (Table 3).

**Table 3:** Stepwise Hierarchical Modeling Results for DD∗

Model	Determinant Factors	*P* Value†	R^2^ (%)	ΔR^2^ (%)
1	Base model (age, age^2^, sex)	N.A.	7.80	N.A.
2	Model 1 + BMI, waist circumference, hip circumference, waist-to-hip ratio, and interaction terms‡	**<2 × 10^–6^**	8.20	0.35
3	Model 2 + arthritis, osteoarthritis, and anti-inflammatory and antirheumatic products	**5.4 × 10^–8^**	8.52	0.32
4	Model 3 + HDL, and triglycerides	**.024**	8.59	0.067
5	Model 4 + (moderate and heavy) alcohol use	**.003**	8.70	0.11
6	Model 5 + diabetes and diabetes drugs	**.039**	8.76	0.059
7	Model 6 + SBP, DBP and MAP	>.05	n.d.	n.d.
8	Model 6 + (ex-)smoking	>.05	n.d.	n.d.
9	Model 6 + ASAT	>.05	n.d.	n.d.

N.A., not applicable; n.d., not determined.

∗ Blocks have been sorted based on increasing significance of the most significant variable within the block (see Table 2).

† *P* values from the log-likelihood ratio test. Significant *P*-values (<.05) are indicated in bold.

‡ Interaction terms = waist circumference × sex, hip circumference × sex, waist-to-hip ratio × sex, and BMI × sex.

Sensitivity analysis using controls aged >55 years showed similar results, with joint related factors being the most highly associated after age and sex. Furthermore, lipid related factors did not contribute significantly to the final model anymore, whereas (ex-)smoking now did (Appendix S1, Tables S3 and S4, available online on the Journal’s website at https://www.jhsgo.org).

## Discussion

In the Lifelines cohort, with a total of 1,320 DD cases (2.1%), we found that age, age^2^, and sex were the major risk factors for DD accounting for 7.8% of the variance in DD presence. Other risk factors for DD were arthritis, osteoarthritis, use of anti-inflammatory or antirheumatic products, alcohol use, higher HDL levels, diabetes, and use of diabetes medication, whereas anthropometric measures of adiposity (including interactions with sex) were associated with lower DD risk. Their contribution was however relatively small, with the explained variance increasing only to 8.8%. In our sensitivity analysis, (ex-)smoking significantly increased the risk of having DD as well, whereas lipid related factors were not significantly associated anymore.

Kang et al^18^ (2024) performed a similar study as ours using the UK Biobank. Our results are mostly consistent with their findings. However, in contrast to theirs, we found significant associations with rheumatoid arthritis and the waist-to-hip-ratio. We could not replicate their associations of hemoglobin A1c that increased the odds of DD, which might be explained by the greater power of the UK Biobank study.^18^

Like others, we observed that higher age and male sex were associated with an increased risk of having DD.^3^^,^^13^^,^^18^^,^^26^, ^27^, ^28^, ^29^, ^30^, ^31^, ^32^, ^33^ Age^2^ was significantly negatively associated with DD presence, implying that DD risk attenuates with increasing age. We found no interaction between age and sex; thus, the effect of age on DD risk is equal for males and females. This aligns with previous studies that reported that DD prevalence increases with age independently from sex.^34^ The predominance in men is in line with the clinical view of male sex being a risk factor for more aggressive disease.^34^, ^35^, ^36^ However, the influence of sex in DD etiology is currently unknown.

We furthermore found that anthropometric indices protect against DD, in line with previous studies.^16^, ^17^, ^18^^,^^30^^,^^31^ The addition of anthropometric variables significantly improved the multivariable model, but the contribution was minor with just 0.35%. This relation might be attributed to the shared effects of genetic variants as a negative genetic correlation was found between BMI and DD previously.^37^ A Mendelian Randomization study showed that genetically predicted higher BMI was associated with a lower risk of getting DD, indicating a causal role of lower BMI on DD.^17^ Interestingly, the interaction terms of anthropometric variables with sex were also negatively associated with DD. This means that the negative association between the anthropometric variables and DD is dependent on sex, with stronger protective effects noted in males than in females. Reasons behind this association are unclear, although an increase in body fat could lead to overgrowth of the palmar fascia based on a decreased level of testosterone.^17^ The latter, however, contradicts the hypothesis that testosterone might stimulate fibroblast proliferation by targeting androgen receptors in the palmar fascia, leading to increased DD prevalence in men.^38^

After age, sex, and anthropometric traits, we found rheumatoid arthritis, osteoarthritis, and the use of anti-inflammatory and antirheumatic products to be most significantly positively associated with having DD (ORs 1.34, 1.34, and 1.46, respectively). This contrasts with findings from other studies. Two small studies observed a lower DD prevalence in individuals with rheumatoid arthritis.^39^^,^^40^ However, studies of higher quality including Kang et al^18^ (2024) did not find an association between rheumatoid arthritis and DD.^41^ Perhaps genetic and immunological factors or medication play a role. Further studies are needed to elucidate this relationship.

Smoking variables did not significantly improve the multivariable model in our main analysis. This contradicts some previous studies that identified cigarette smoking as a risk factor for DD.^13^^,^^18^^,^^30^^,^^32^^,^^42^, ^43^, ^44^ However, after repeating our analyses including only controls aged >55 years, (ex-)smoking significantly increased the risk of having DD. This aligns with previous literature describing a dose–response relationship with the highest DD risk for heavy smokers and suggests that cigarette smoking might cause microvascular occlusive disease leading to the development of fibrotic contractures.^30^^,^^42^^,^^44^^,^^45^ However, other studies found weak associations between smoking and DD presence or were inconclusive.^27^^,^^33^^,^^46^^,^^47^ Current smoking was not significantly associated with DD. This discrepancy might have been caused by population differences, the level of adjustment for confounding, or used measurement methods.

Heavy alcohol use has previously been described to increase DD risk.^13^^,^^18^^,^^33^^,^^44^ In our study, both moderate and heavy alcohol drinking were significantly associated with DD. Heavy drinkers had a higher DD risk than moderate drinkers compared to nondrinkers, suggesting a dose–response effect.

Using the Lifelines cohort this study employed a large sample size from the general population, meaning that our results are generalizable. Additionally, several interaction terms were taken into account and robustness of our findings was determined by a sensitivity analysis. Furthermore, we introduced a wide range of novel predictors such as gallstones, hepatitis, osteoarthritis, apolipoprotein A1 and B100 levels, ASAT levels, ALAT levels, and many medication types, especially anti-inflammatory and antirheumatic drugs. However, a first limitation of this study is that we did not perform a systemic review to find all the relevant phenotypic factors for inclusion in this study. Thus, some risk factors might be missing in our modeling strategy. Furthermore, our data source did not capture relevant data, such as medication dosing, compliance, or the indications for use as well as measures of manual work or vibration exposure, which prevented us from including these in our analysis. Second, it is known that DD is underdiagnosed. A previous study reported that 20% of the population over the age of 50 years show signs of DD.^27^ Many Lifelines controls may therefore unknowingly have DD, resulting in obscured associations with DD and deflated percentage of explained variance. However, although not all subjects filled out the questionnaire, the effect of nonresponse bias was limited (Table S5, available online on the Journal’s website at https://www.jhsgo.org). Third, DD in this study was determined by the question whether DD was ever diagnosed by a doctor. Time of the diagnosis is unknown. Therefore, we could only investigate associations and not causal relationships prohibiting the creation of a prediction model.

In summary, next to known risk factors, we found significant associations of rheumatoid arthritis, osteoarthritis, and anti-inflammatory and antirheumatic drugs with DD. Physicians should be more careful in prescribing these drugs to individuals at high risk for DD (eg, high genetic load or family members with DD). The total contribution of identified risk factors was however only modest. Integrating genetic risk factors will likely further improve the risk model because many genetic risk factors have already been identified for DD.^48^ This might enable the generation of highly accurate models ultimately leading to personal risk prediction for DD presence.

## Data Availability

Data may be obtained from a third party and are not publicly available. Researchers can apply to use the Lifelines data used in this study. More information about how to request Lifelines data and the conditions of use can be found on their website (https://www.lifelines-biobank.com/researchers/working-with-us).

## Conflicts of Interest

Prof Dr Werker is a consultant for FIDIA-IT and Novotech-AU. Remunerations are used for research purposes only. No benefits in any form have been received or will be received related directly to this article. None of the authors has a financial interest in the work presented in this article.
