# Supplementary material for: Associations of Demographic, Lifestyle, and Clinical Factors With the Presence of Dupuytren Disease: Results from the Lifelines Cohort Study
Source: J Hand Surg Glob Online. 2025 Jul 24;7(5):100786. doi: 10.1016/j.jhsg.2025.100786 (PMC12309943; doi:10.1016/j.jhsg.2025.100786)
Supplement: Supplementary Material [file mmc1.docx]

**Supplementary Data to “Associations of demographic, lifestyle and clinical factors with the presence of Dupuytren’s disease: results from the Lifelines Cohort Study”**

Michel F. N. Noordman^1,2^, Sophie A. Riesmeijer^1,2^, Paul M. N. Werker^1^, Ilja M. Nolte^2^

^1^University of Groningen, University Medical Center Groningen, Department of Plastic

Surgery, Hanzeplein 1, 9713 GZ, Groningen, The Netherlands

^2^University of Groningen, University Medical Center Groningen, Department of

Epidemiology, Hanzeplein 1, 9713 GZ , Groningen, The Netherlands

**Contents**

Appendix S1. Results of the sensitivity analysis 2

Table S1. Details of the selected demographic, lifestyle and clinical variables

including the measurement method and the visit at which it was measured. 3

Table S2. Patient characteristics stratified for DD patients and controls, the latter

with an age at visit 3a ≥55 year. 6

Table S3. Univariable logistic regression analyses results for sensitivity analysis

without (unadjusted OR and p-value) and with (adjusted OR and p-value) correction

for age, age^2^, and sex. 9

Table S4. Stepwise hierarchical modeling results for the sensitivity analysis. 11

Table S5. Non-response bias analysis. 12

**Appendix S1**

*Results of the sensitivity analysis*

After applying the cutoff of ≥55 years to the control group, being an ex-smoker increased the odds of having DD compared to never smokers (OR=1.14 [1.01-1.29]; P=0.03) in the age and sex adjusted analysis (Table S3). Additionally, the lipemia index (OR=0.99 [0.98-1.00] P=0.09) now had a P-value below our significance threshold for inclusion in the multivariable models. Conversely, ASAT was not associated anymore with the presence of DD (P=0.41).

For the sensitivity analysis, the composition of the blocks of variables were more or less the same as in the main analysis, with the exception of inclusion of lipemia index in the lipid-related block, and the removal of ASAT. However, the order in which the models were tested hierarchically differed, as can be seen in Table S4. The explained variance of the base model was substantially reduced to only 4.32%, as expected, since the effect of age was mostly taken out (Table S4). Most notably, the joint-related variables were the most highly associated with DD after age and sex as opposed to the main analysis, raising the explained variance to 4.66%. The inclusion of the anthropometric measures BMI, waist- and hip circumference, waist-to-hip ratio, and the interaction terms (model 2) increased the amount of explained variance even more (0.43%) compared to the main analysis (0.35%). Inclusion of moderate and heavy alcohol use to model 3 now explained 0.15% of the variance compared to 0.11% earlier. Adding diabetes and diabetes drugs explained slightly more of the variance with an increase of 0.09%. Models 6 and 7 did not contribute significantly to the explained variance, whilst in our main analyses lipid-related variables still contributed significantly. Moreover, the explained variance after adding ex-smoking, which did not contribute significantly to the final model in our main analysis, now became slightly more (0.08%). The final model was therefore model 8, which explained a total of 5.39% of the individual differences in the presence of DD.

**Table S1.** Details of the selected demographic, lifestyle and clinical variables including the measurement method and the visit at which it was measured.

| **Characteristic** | **Visit^*^** | **Measurement method** |
| --- | --- | --- |
| **Demographic variables** |  |  |
| Age (years) | 3A | Questionnaire |
| Sex | N.A. | Self-reported biological sex |
| **Anthropometric variables** |  |  |
| BMI (kg/m^2^) | 3A | (weight/((height/100)^2^)), measured without shoes, wearing light clothing with empty pockets |
| Waist circumference (cm) | 3A | SECA 201 measurement tape placed between lowest rib and the ilical crest around the bare stomach in upright position. |
| Hip circumference (cm) | 3A | SECA 201 measurement tape is placed at the widest part of the buttocks in upright position |
| Waist-to-hip ratio | 3A | Waist circumference/hip circumference |
| **Diabetes-related variables** |  |  |
| Diabetes mellitus | 1A/1B/ 1C/2A | Type 1 or 2 diabetes at either visit, based on self-reported diabetes, diabetes medication use (only for 1A), fasting plasma glucose ≥ 7.0 mmol/L (only for 1A and 2A) or HbA1c ≥ 6.5 (only for 1A and 2A) |
| Diabetes drugs | 1A | ATC code A10 |
| HbA1c (mmol/mol) | 3A | BD Vacutainer® 4.0 mL K2E (EDTA) 7.2mg Plus blood collection tubes |
| Glucose (mmol/L) | 3A | BD Vacutainer® 4.0 mL NaF 3.0mg Na2EDTA 6.0mg Plus blood collection tubes |
| **Lipid-related variables** |  |  |
| Low-density lipoprotein  (mmol/L) | 3A | BD Vacutainer® 4.5 mL LH (Lithium Heparin) PST™ II Plus blood collection tubes |
| High-density lipoprotein  (mmol/L) | 3A | BD Vacutainer® 4.5 mL LH (Lithium Heparin) PST™ II Plus blood collection tubes |
| Total cholesterol (mmol/L) | 3A | BD Vacutainer® 4.5 mL LH (Lithium Heparin) PST™ II Plus blood collection tubes |
| Triglycerides (mmol/L) | 3A | BD Vacutainer® 4.5 mL LH (Lithium Heparin) PST™ II Plus blood collection tubes |
| Lipemia index | 3A | N.A. |
| Apolipoprotein A1 (g/L) | 1A | BD Vacutainer® 4.5 mL LH (Lithium Heparin) PST™ II Plus blood collection tubes |
| Apolipoprotein B100 (g/L) | 1A | BD Vacutainer® 4.5 mL LH (Lithium Heparin) PST™ II Plus blood collection tubes |
| Lipid modifying agents | 1A | ATC code C10 |
| Anti-thrombotic agents | 1A | ATC code B01 |
| **Blood pressure-related variables** | | |
| SBP (mmHg) | 1A | DinaMap PRO100 or DinaMap PRO100V2. Average of three measurements in sitting position. Corrected for blood pressure lowering medication use at baseline: +15 mmHg |
| DBP (mmHg) | 1A | DinaMap PRO100 or DinaMap PRO100V2. Average of three measurements in sitting position. Corrected for blood pressure lowering medication use at baseline: +10 mmHg |
| MAP | N.A. | Calculated as 2/3*SBP – 1/3*DBP |
| RAAS-acting agents | 1A | ATC code C09 |
| Calcium channel blockers | 1A | ATC code C08 |
| Diuretics | 1A | ATC code C03 |
| Other antihypertensives | 1A | ATC code C02 |
| **Smoking variables** |  |  |
| Pack years | 1A | Cumulative smoking history: 1 packyear = 20 cigarettes per day for 1 year (or 10 cigarettes for 2 years, or 1 cigarette for 20 years). NB. Cigars are regarded as 3 cigarettes. |
| Smoking categorical (never, ex, current smoker) | All | Derived from questionnaire data: Never, ex, current, or recent smoker. Recent was coded as current by us. |
| **Alcohol intake variables** |  |  |
| Alcohol use (not, moderate, heavy) | All | How often did you drink alcoholic drinks (including non-alcoholic beer) in the past month (not this month, 1 day/month, 2-3 days/month, 1 day/week, 2-3 days/week, 4-5 days/week, 6-7 days/week)? AND How many glasses did you drink on average on such a day? Using this data ‘drinking days per month was created’. Using ‘glasses per day’ we calculated ‘glasses per week’ Thereafter, the NHS cutoff for alcohol consumption was applied. |
| **Joint-related variables** |  |  |
| Rheumatoid arthritis | All | Baseline: could you indicate if you have (had) rheumatoid arthritis (joint inflammation)?  Follow up visits: did rheumatoid arthritis start since the last time you filled out the Lifelines questionnaire? |
| Osteoarthritis | All | Baseline: could you indicate if you have (had) osteoarthritis (joint degradation)?  Follow up visits: did osteoarthritis start since the last time you filled out the Lifelines questionnaire? |
| Anti-inflammatory and anti-rheumatic drugs | 1A | ATC code M01 |
| Antigout preparations | 1A | ATC code M04 |
| Immunostimulants | 1A | ATC code L03 |
| Immunosuppressants | 1A | ATC code L04 |
| **Gastrointestinal and liver-related variables** | | |
| Gallstones | All | Baseline: could you indicate if you have (had) gallstones?  Follow up visits: did gallstones start since the last time you filled out the Lifelines questionnaire? |
| Hepatitis | All | Baseline: could you indicate if you have (had) hepatitis?  Follow up visits: did hepatitis start since the last time you filled out the Lifelines questionnaire? |
| Liver disorder | All | Baseline: could you indicate if you have (had) liver cirrhosis?  Follow up visits: did a liver disorder start since the last time you filled out the Lifelines questionnaire? |
| ALAT (u/L) | 1A | BD Vacutainer® 4.5 mL LH (Lithium Heparin) PST™ II Plus blood collection tubes |
| ASAT (u/L) | 1A | BD Vacutainer® 4.5 mL LH (Lithium Heparin) PST™ II Plus blood collection tubes |
| ****Other variables** |  |  |
| Lung fibrosis | 1C | Have you ever been told by a doctor that you had scarring of the lungs (pulmonary fibrosis)? |

BMI, body mass index; SBP, systolic blood pressure; DBP, diastolic blood pressure; MAP, mean arterial pressure; HbA1c, hemoglobin A1c; RAAS, renin-angiotensin-aldosterone system; ALAT, alanine aminotransferase; ASAT, aspartate aminotransferase; N.A., not applicable.

^*^ ‘All’ means that data of visit 3B or 3A was considered first, and when no data were available at these visits, data were taken from earlier visits going back in time. For medication use, individuals with missing data at all visits were considered non-users. Wave 1A is the baseline visit, 1B ~3 years after baseline, 1C ~5 years after baseline, 2A ~7 years after baseline, 2B ~9.5 years after baseline, 3A ~12 years after baseline, 3B ~14.5 years after baseline.

**The term ‘Other variables’ refers to variables that did not fit in one of the other group of variables.

**Table S2.** Characteristics stratified for DD patients and controls, the latter with an age at visit 3a ≥55 year. Presence of DD was determined by a positive answer to the question whether he/she was diagnosed by a doctor. Controls are the participants (older than 55 years) who reported not to have a positive doctor’s diagnosis of DD.

| **Characteristic** | **DD (n=1,320)^#^** | **No DD (n=31,991)^#^** |
| --- | --- | --- |
| **Demographic variables** |  |  |
| Age, years | 64.3 (9.2) | 64.5 (6.9) |
| Sex (male) | 746 (57%) | 13,741 (43%) |
| **Anthropometric variables** |  |  |
| BMI (kg/m^2^) | 25.9 [23.7-28.6] | 26.4 [24.0-29.2] |
| Waist circumference (cm) | 93.0 [86.0-101.0] | 93.0 [85.0-101.5] |
| Hip circumference (cm) | 100.0 [95.5-105.0] | 101.0 [96.0-106.0] |
| Waist-to-hip ratio | 0.93 [0.86-0.99] | 0.92 [0.86-0.98] |
| **Diabetes-related variables** |  |  |
| Diabetes  No  Yes | 1,228 (93.2%)  90 (6.8%) | 30,225 (94.5%)  1,744 (5.5%) |
| Diabetes drugs  No  Yes | 1,274 (96.5%)  46 (3.5%) | 31,353 (98.0%)  638 (2.0%) |
| HbA1c (mmol/mol) | 38.0 [36.0-40.0] | 38.0 [36.0-41.0] |
| Glucose (mmol/L) | 5.3 [5.0-5.8] | 5.3 [5.0-5.7] |
| **Lipid-related variables** |  |  |
| LDL cholesterol^*^ | 3.43 (0.94) | 3.50 (0.95) |
| HDL cholesterol^*^ | 1.50 [1.20-1.80] | 1.50 [1.20-1.80] |
| Total cholesterol (mmol/L)^*^ | 5.3 (1.04) | 5.4 (1.06) |
| Triglycerides (mmol/L) | 1.11 [0.84-1.50] | 1.14 [0.86-1.57] |
| Lipemia index | 10.0 [8.0-13.0] | 11.0 [9.0-13.0] |
| Apolipoprotein A1 (g/L) | 1.55 (0.26) | 1.56 (0.26) |
| Apolipoprotein B100 (g/L) | 0.97 (0.25) | 0.97 (0.23) |
| Lipid modifying agents  No  Yes | 1,193 (90.4%)  127 (9.6%) | 29,111 (91.0%)  2,880 (9.0%) |
| Antithrombotic agents  No  Yes | 1,240 (93.9%)  80 (6.1%) | 30,331 (94.8%)  1,660 (5.2%) |
| **Blood pressure variables** |  |  |
| SBP (mmHg)^*^ | 128.0 [118.0-141.0] | 129.0 [118.0-141.0] |
| DBP (mmHg)^*^ | 77.0 [69.0-84.0] | 76.0 [69.0-84.0] |
| MAP^*^ | 94.0 [86.3-102.0] | 93.7 [86.3-102.3] |
| RAAS-acting agents  No  Yes | 1,193 (90.4%)  127 (9.6%) | 28,903 (90.3%)  3,088 (9.7%) |
| Calcium channel blockers  No  Yes | 1,277 (96.7%)  43 (3.3%) | 31,132 (97.3%)  859 (2.7%) |
| Diuretics  No  Yes | 1,255 (95.1%)  65 (4.9%) | 30,348 (94.9%)  1,643 (5.1%) |
| Other antihypertensives  No  Yes | 1,318 (>99.3%)  <10 (<0.7%) | 31,939 (99.8%)  52 (0.2%) |
| **Smoking variables** |  |  |
| Packyears | 3.0 [0.0-12.2] | 2.3 [0.0-12.0] |
| Smoking  Never  Ex  Current | 496 (37.7%)  691 (52.5%)  129 (9.8%) | 12,286 (38.5%)  16,220 (50.8%)  3,414 (10.7%) |
| **Alcohol intake variables** |  |  |
| Alcohol  Not  Moderate  Heavy | 249 (18.9%)  972 (73.7%)  97 (7.4%) | 7,464 (23.4%)  22,913 (71.8%)  1542 (4.8%) |
| **Joint-related variables** |  |  |
| Arthritis  No  Yes | 1,229 (93.1%)  91 (6.9%) | 30,206 (94.4%)  1,785 (5.6%) |
| Osteoarthritis  No  Yes | 860 (65.2%)  460 (34.8%) | 21,867 (68.4%)  10,124 (31.6%) |
| Anti-inflammatory and anti-rheumatic products  No  Yes | 1,237 (93.7%)  83 (6.3%) | 30,479 (95.3%)  1,512 (4.7%) |
| Antigout preparations  No  Yes | 1,314 (>99.3%)  <10 (<0.7%) | 31,856 (99.6%)  135 (0.4%) |
| Immunostimulants  No  Yes | 1,320 (>99.3%)  <10 (<0.7%) | 31,977 (99.96%)  14 (0.04%) |
| Immunosuppressants  No  Yes | 1,313 (>99.3%)  <10 (<0.7%) | 31,796 (99.4%)  195 (0.6%) |
| **Gastrointestinal and liver-related variables** | | |
| Gallstones  No  Yes | 1,259 (95.4%)  61 (4.6%) | 30,222 (94.5%)  1,769 (5.5%) |
| Hepatitis  No  Yes | 1,294 (98.0%)  26 (2.0%) | 31,426 (98.2%)  565 (1.8%) |
| Liver disorder  No  Yes | 1,310 (>99.3%)  <10 (<0.7%) | 31,786 (99.4%)  205 (0.6%) |
| ALAT (u/L) | 21.0 [16.0-28.0] | 20.0 [15.0-28.0] |
| ASAT (u/L) | 24.0 [20.0-27.0] | 23.0 [20.0-28.0] |
| ****Other variables** |  |  |
| Lung fibrosis  No  Yes | 1,060 (>99.2%)  <10 (<0.8%) | 25,582 (99.0%)  249 (1.0%) |

DD, Dupuytren disease; BMI, body mass index; LDL, low-density lipoprotein; HDL, high-density lipoprotein; SBP, systolic blood pressure; DBP, diastolic blood pressure; MAP, mean arterial pressure; HbA1c, hemoglobin A1c; RAAS, renin-angiotensin-aldosterone system; ALAT, alanine aminotransferase; ASAT, aspartate aminotransferase.

^*^ adjusted for medication use (see Methods).

** the term ‘Other variables’ refers to variables that did not fit in one of the other group of variables.

^#^ continuous variables are described using mean (standard deviation) if normally distributed or median [interquartile range] otherwise; categorical data are described as count (percentage).

**Table S3.** Univariable logistic regression analyses results for sensitivity analysis without (unadjusted OR and p-value) and with (adjusted OR and p-value) correction for age, age^2^, and sex. Interaction terms were only tested in models corrected for age, age^2^, and sex. Significant p-values (p<0.05) are indicated in bold, p-value <0.25 in italic. The number after the name of the block of variables indicates the order of significance as used for the multivariable, hierarchical modelling, with no number meaning that none of the individual variables was significant.

| **Variable** | **Unadjusted** | | **Adjusted** | |
| --- | --- | --- | --- | --- |
|  | **OR (95% CI)** | **P-value** | **OR (95% CI)** | **P-value** |
| **Demographic - 1** |  |  |  |  |
| Age | 0.995 (0.987, 1.003) | *0.20* | N.A. | N.A. |
| Age  Age^2^ | 0.862 (0.847, 0.876)  1.006 (1.005, 1.007) | **<2x10^-16^**  **<2x10^-16^** | N.A.  N.A. | N.A.  N.A. |
| Sex (male) | 1.726 (1.545, 1.929) | **<2x10^-16^** | N.A. | N.A. |
| Age*sex | N.A. | N.A. | 0.999 (0.985, 1.012) | 0.84 |
| **Anthropometric - 3** |  |  |  |  |
| BMI | 0.969 (0.956, 0.983) | **9.2x10^-6^** | 0.968 (0.954, 0.982) | **8.0x10^-6^** |
| BMI*sex | N.A. | N.A | 0.958 (0.930, 0.986) | **0.004** |
| Waist circumference | 1.000 (0.996, 1.005) | 0.97 | 0.992 (0.986, 0.997) | **0.001** |
| Waist circumference*sex | N.A. | N.A. | 0.988 (0.978, 0.998) | **0.02** |
| Hip circumference | 0.988 (0.981, 0.994) | **0.0003** | 0.990 (0.983, 0.997) | **0.007** |
| Hip circumference*sex | N.A. | N.A | 0.986 (0.972, 1.000) | *0.06* |
| Waist-to-hip ratio | 2.780 (1.501, 5.136) | **0.001** | 0.372 (0.164, 0.842) | **0.02** |
| Waist-to-hip ratio*sex | N.A. | N.A | 0.148 (0.029, 0.751) | **0.02** |
| **Diabetes-related - 5** |  |  |  |  |
| Diabetes | 1.270 (1.013, 1.572) | **0.03** | 1.221 (0.969, 1.519) | *0.08* |
| Diabetes drugs | 1.774 (1.292, 2.378) | **2x10^-4^** | 1.732 (1.252, 2.339) | **6x10^-4^** |
| HbA1c | 0.997 (0.986, 1.007) | 0.55 | 0.999 (0.988, 1.009) | 0.80 |
| Glucose | 1.039 (0.981, 1.098) | *0.18* | 1.005 (0.946, 1.064) | 0.87 |
| **Lipid-related - 6** |  |  |  |  |
| LDL cholesterol^*^ | 0.926 (0.872, 0.984) | **0.01** | 0.968 (0.911, 1.029) | 0.30 |
| HDL cholesterol^*^ | 0.938 (0.822, 1.070) | 0.34 | 1.268 (1.094, 1.468) | **0.001** |
| Total cholesterol^*^ | 0.917 (0.868, 0.968) | **0.002** | 0.983 (0.929, 1.039) | 0.55 |
| Lipemia index | 0.997 (0.987, 1.006) | 0.53 | 0.992 (0.982, 1.001) | *0.09* |
| Triglycerides | 0.950 (0.874, 1.028) | *0.21* | 0.927 (0.852, 1.003) | *0.07* |
| Apolipoprotein A1 | 0.867 (0.567, 1.316) | 0.51 | 1.302 (0.819, 2.052) | 0.26 |
| Apolipoprotein B100 | 0.921 (0.568, 1.484) | 0.74 | 0.988 (0.601, 1.613) | 0.96 |
| Lipid modifying agents | 1.076 (0.889, 1.292) | 0.44 | 1.014 (0.831, 1.228) | 0.89 |
| Antithrombotic agents | 1.179 (0.928, 1.476) | *0.16* | 0.940 (0.730, 1.194) | 0.62 |
| **Blood pressure-related - 7** | | | | |
| SBP^*^ | 0.999 (0.996, 1.002) | 0.59 | 0.995 (0.992, 0.999) | **0.006** |
| DBP^*^ | 1.001 (0.995, 1.006) | 0.80 | 0.995 (0.990, 1.001) | *0.12* |
| MAP^*^ | 1.000 (0.995, 1.004) | 0.91 | 0.994 (0.989, 0.999) | **0.02** |
| RAAS-acting agents | 0.996 (0.823, 1.196) | 0.97 | 0.945 (0.774, 1.143) | 0.57 |
| Calcium channel blockers | 1.220 (0.881, 1.645) | *0.21* | 1.088 (0.777, 1.482) | 0.61 |
| Diuretics | 0.957 (0.735, 1.223) | 0.73 | 0.970 (0.739, 1.252) | 0.82 |
| Other antihypertensives | 0.932 (0.152, 2.999) | 0.92 | 0.892 (0.144, 2.930) | 0.88 |
| **Smoking variables - 8** |  |  |  |  |
| Packyears | 1.001 (0.996, 1.006) | 0.70 | 1.001 (0.995, 1.006) | 0.82 |
| Smoking  Never  Ex  Current | Ref.  1.055 (0.938, 1.188)  0.936 (0.765, 1.136) | Ref.  0.37  0.51 | Ref.  1.144 (1.013, 1.291)  0.934 (0.761, 1.138) | Ref.  **0.03**  0.51 |
| **Alcohol intake - 4** |  |  |  |  |
| Alcohol  Not  Moderate  Heavy | Ref.  1.272 (1.106, 1.468)  1.886 (1.476, 2.391) | Ref.  **8x10^-4^**  **2.5x10^-7^** | Ref.  1.200 (1.040, 1.390)  1.580 (1.227, 2.021) | Ref.  **0.014**  **3x10^-4^** |
| **Joint-related - 2** |  |  |  |  |
| Arthritis | 1.253 (1.001, 1.549) | **0.04** | 1.341 (1.068, 1.664) | **0.009** |
| Osteoarthritis | 1.155 (1.028, 1.296) | **0.01** | 1.366 (1.210, 1.540) | **4.3x10^-7^** |
| Anti-inflammatory and anti-rheumatic products | 1.353 (1.069, 1.688) | **0.009** | 1.477 (1.165, 1.848) | **9x10^-4^** |
| Antigout preparations | 1.077 (0.422, 2.238) | 0.86 | 0.744 (0.286, 1.586) | 0.49 |
| Immunostimulants | 3.11x10^-5^ (0, 0.460) | 0.94 | 3.28x10^-5^ (0, 0.447) | 0.94 |
| Immunosuppressants | 0.869 (0.369, 1.714) | 0.72 | 0.892 (0.377, 1.767) | 0.77 |
| **Gastrointestinal and liver-related variables - 9** | | | | |
| Gallstones | 0.828 (0.631, 1.065) | *0.16* | 0.951 (0.722, 1.229) | 0.71 |
| Hepatitis | 1.118 (0.733, 1.627) | 0.58 | 1.259 (0.822, 1.841) | 0.26 |
| Liver disorder | 1.184 (0.585, 2.120) | 0.60 | 1.288 (0.635, 2.314) | 0.44 |
| ALAT | 1.002 (0.997, 1.006) | 0.28 | 1.000 (0.994, 1.005) | 0.92 |
| ASAT | 1.000 (0.988, 1.012) | 0.96 | 0.995 (0.981, 1.007) | 0.41 |
| ****Other variables** |  |  |  |  |
| Lung fibrosis | 0.969 (0.480, 1.732) | 0.92 | 0.972 (0.480, 1.742) | 0.93 |

DD, Dupuytren disease; OR, odds ratio; CI, confidence interval; N.A., not applicable; BMI, body mass index; LDL, low-density lipoprotein; HDL, high-density lipoprotein; SBP, systolic blood pressure; DBP, diastolic blood pressure; MAP, mean arterial pressure; HbA1c, hemoglobin A1c; Ref., reference category; ALAT, alanine aminotransferase; ASAT, aspartate aminotransferase.

^*^ adjusted for cholesterol lowering medication use.

** the term ‘Other variables’ refers to variables that did not fit in one of the other group of variables.

**Table S4.** Stepwise hierarchical modeling results for the sensitivity analysis. Blocks have been sorted based on increasing significance of the most significant variable within the block (see table S3).

| **Model** | **Determinant factors** | **P-value^#^** | **R^2^ (%)** | **ΔR^2^ (%)** |
| --- | --- | --- | --- | --- |
| 1 | Base model (age, age^2^, and sex) | N.A. | 4.32 | N.A. |
| 2 | Model 1 + arthritis, osteoarthritis, and anti-inflammatory and anti-rheumatic drugs | **3.5x10^-7^** | 4.66 | 0.34 |
| 3 | Model 2 + BMI, waist circumference, hip circumference, waist-to-hip ratio, and interaction terms* | **3.1x10^-6^** | 5.09 | 0.43 |
| 4 | Model 3 + (moderate and heavy) alcohol use | **7.9x10^-4^** | 5.23 | 0.15 |
| 5 | Model 4 + diabetes and diabetes drugs | **0.02** | 5.31 | 0.09 |
| 6 | Model 5 + lipemia index, triglycerides, and HDL | >0.05 | n.d. | n.d. |
| 7 | Model 5 + SBP, DBP and MAP | >0.05 | n.d. | n.d. |
| 8 | Model 5 + (ex-)smoking | **0.02** | 5.39 | 0.08 |

BMI, body mass index; HDL, high-density lipoprotein; SBP, systolic blood pressure; DBP, diastolic blood pressure; MAP, mean arterial pressure.

*Interaction terms = waist circumference*sex, hip circumference*sex, waist-to-hip ratio*sex, and BMI*sex.

^#^P-value from the log-likelihood ratio test. Significant p-values (p<0.05) are indicated in bold.

**Table S5**. Non-response bias analysis.

| **Baseline data** | **Included** | **Excluded** |
| --- | --- | --- |
| N | 62,941 (37.5%) | 105,087 (62,5%) |
| Age | 45.8 (11.7) | 43.8 (14.0) |
| Gender (females) | 35,622 (58.5%) | 52,836 (58.5%) |
| BMI | 25.6 (4.2) | 25.6 (4.9) |
| Height | 174.8 (9.6) | 173.1 (11.3) |
| Weight | 78.5 (15.3) | 77.4 (18.1) |
| Waist circumference | 89.1 (12.2) | 88.6 (14.2) |
| Hip circumference | 98.7 (9.6) | 98.0 (11.8) |
| Waist-hip ratio | 0.90 (0.08) | 0.90 (0.09) |
| SBP* | 126.1 (16.4) | 126 (18.2) |
| DBP* | 74.5 (10.3) | 73.6 (11.1) |
| LDL* | 3.3 (0.9) | 3.3 (1.0) |
| HDL* | 1.5 (0.4) | 1.5 (0.4) |
| Total cholesterol* | 5.2 (1.0) | 5.1 (1.1) |
| Triglycerides | 1.1 (0.7) | 1.2 (0.8) |
| Diabetes | 2,219 (3.5%) | 4,677 (4.4%) |
| Glucose | 5.0 (0.8) | 5.0 (0.9) |
| HbA1c | 5.5 (0.4) | 5.5 (0.5) |
| Smoking status |  |  |
| *never* | 28,311 (46.7%) | 38,604 (46.7%) |
| *ex* | 21,908 (36.1%) | 27,139 (31.5%) |
| *current* | 10,399 (17.2%) | 20,521 (23.8%) |
| Packyears | 5.7 (9.2) | 6.5 (10.4) |
| Alcohol use |  |  |
| *not* | 11,386 (20.1%) | 17,783 (22.4%) |
| *moderate (0-14 glasses/week)* | 40,824 (72.0%) | 54,957 (69.3%) |
| *heavy (>14 glasses/week)* | 4,499 (7.9%) | 6,524 (8.2%) |
| Number of glasses of alcohol/week | 5.5 (6.8) | 5.3 (7.1) |
| Arthritis | 1,154 (1.9%) | 1,908 (2.1%) |
| Osteoarthritis | 4,557 (7.5%) | 6,384 (7.1%) |

BMI, body mass index; SBP, systolic blood pressure; DBP, diastolic blood pressure; LDL, low-density lipoprotein; HDL, high-density lipoprotein; HbA1c, hemoglobin A1c.
